# Prevalence of Toxoplasma gondii IgG Antibodies in Psychiatric Patients from Western Romania

**DOI:** 10.1192/j.eurpsy.2024.710

**Published:** 2024-08-27

**Authors:** S. Grada, M. A. Lupu, D. A. Oatis, A. G. Mihu, T. R. Olariu

**Affiliations:** ^1^County Emergency Hospital Arad, Arad; ^2^ Discipline of Parasitology; ^3^Center for Diagnosis and Study of Parasitic Diseases, Victor Babes University of Medicine and Pharmacy, Timisoara, Romania

## Abstract

**Introduction:**

*Toxoplasma gondii*, a ubiquitous protozoan parasite, has been previously associated with psychiatric disorders.

**Objectives:**

To assess the prevalence of IgG antibodies against *T. gondii* in psychiatric patients from Western Romania.

**Methods:**

We included 464 psychiatric patients admitted to the Psychiatric Clinic, County Emergency Hospital of Arad, Western Romania. Clinical evaluation and laboratory tests were conducted in these patients, including serological tests to determine the presence of *T. gondii* IgG antibodies.

**Results:**

Of the 464 psychiatric patients, 258 (55.5%) were residing in rural areas and 245 (52.7%) were female. *T. gondii* IgG antibodies were demonstrated in 325 (70.04%) of 464 study participants and the seroprevalence tended to increase with age.

A significant higher *T. gondii* IgG seroprevalence was found in psychiatric patients aged between 40 to 59 years (p<0.001) and in patients aged ≥60 years (p=0.001) compared to patients aged 19 to 39 years. A higher *T. gondii* IgG seroprevalence was determined in psychiatric patients residing in rural areas compared to those residing in urban areas (p=0.04). T. gondii IgG seroprevalence was higher in females compared to males (p=0.04).

Assessment of seroprevalence by diagnostic revealed that *T. gondii* IgG antibodies were identified in 23 (85.19%) of 27 patients with delusional disorders, 24 (82.76%) of 29 with dementia, 51 (70.83%) of 72 with organic disorders, 75 (70.75%) of 106 with schizophrenia, 81 (70.43%) of 115 patients with depression, 17 (62.96%) of 27 with bipolar disorders, 27 (58.7%) of 46 with mood disorders and 7 (53.85%) of 13 with impulsive-control disorders.

**Conclusions:**

The presence of *T. gondii antibodies* was demonstrated in a significant number of patients who attended the Psychiatric Clinic in Arad County, Western Romania. Results of this study suggest that *T. gondii* may be associated with several psychiatric disorders.

**Disclosure of Interest:**

None Declared

